# Knowledge and attitudes about sudden death in epilepsy among people living with epilepsy and their healthcare providers in Mulago Hospital, Uganda: A cross‐sectional study

**DOI:** 10.1002/epi4.12374

**Published:** 2019-12-26

**Authors:** Mark Kaddumukasa, Emmanuel Mwesiga, Nelson Sewankambo, Carol Blixen, Samden Lhatoo, Martha Sajatovic, Elly Katabira

**Affiliations:** ^1^ Department of Medicine College of Health Sciences Makerere University Kampala Uganda; ^2^ Department of Psychiatry College of Health Sciences Makerere University Kampala Uganda; ^3^ Neurological and Behavioral Outcome Center Case Medical Center University Hospitals Cleveland OH USA; ^4^ Department of Neurology Health Science Center at Houston The University of Texas Houston TX USA

**Keywords:** healthcare providers, knowledge, sudden death in epilepsy (SUDEP)

## Abstract

**Objective:**

The objective of the study was to assess level of knowledge and attitudes of SUDEP among people living with epilepsy (PLWE) and healthcare workers providing epilepsy care in Uganda.

**Methods:**

This cross‐sectional study of 48 PLWE and 19 epilepsy care providers used a tailored questionnaire to evaluate epilepsy and SUDEP knowledge, frequency of SUDEP discussion, reasons for not discussing SUDEP, timing of SUDEP discussions, and perceived patient reactions to being provided information on SUDEP.

**Results:**

Median PLWE sample age was 25 (IQR; 19‐34) years, 10 (20.8%) were male, median age of onset of epilepsy 12 (IQR; 6‐18) years. Half of the PLWE reported that they had never heard of SUDEP. Most PLWE desired detailed information regarding SUDEP and preferred this information during the subsequent visits. Healthcare provider sample mean age was 35.7 (22.8) years, 12 (63.2%) were male and composed of 4 physicians (21.1%). Only 15% (3/20) of providers discussed SUDEP with their patients while 85% (17/20) have never discussed it. The main reasons for not discussing SUDEP were not knowing enough about SUDEP (89.5%) and no adequate support network available (30%). Providers that discussed SUDEP (100%) reported that negative reactions were the most common patient response.

**Significance:**

In this Ugandan sample, most PLWE are not aware of SUDEP and epilepsy care providers rarely discuss SUDEP with their patients or patient caregivers. Negative reactions to SUDEP discussions are common but not universal. There is an urgent need for epilepsy educational programs in clinics and targeted communities addressing SUDEP.

**Key points**

- Ugandan epilepsy healthcare providers rarely discuss SUDEP with their patients.
- Majority of PLWE desired detailed information regarding SUDEP.
- Having insufficient information regarding SUDEP was reported to be the main reason for not discussing it.

## 1 INTRODUCTION

Epilepsy affects nearly 70 million people worldwide, with nearly 90% residing in low and middle‐income countries (LMICs).^1^ Sudden unexpected death in epilepsy (SUDEP) is defined as a sudden and unexpected non‐traumatic or non‐drowning–related death in a person with epilepsy which may or may not associate with a recent seizure.^2^ SUDEP remains a leading cause of mortality among people with seizure disorders.^3^

Whereas reports from World Health Organization show that sub‐Saharan Africa shoulders the biggest burden of epilepsy worldwide, information regarding SUDEP is limited and based on small cohorts.^4^, ^5^, ^6^ Recently, a study by Ngugi et al reported a mortality rate of 33.3/1000 persons/year among people with active convulsive epilepsy with an overall standardized mortality ratio of 6.5 which is 6 times higher than that of the general population.^7^

Despite the lack of clarity regarding the mechanisms of SUDEP, several factors have been reported such as frequent seizures, especially generalized tonic‐clonic seizures (GTCS), and anti‐epileptic drug non‐adherence.^7^, ^8^ The AAN/AES guidelines appraised and synthesized most recent literature regarding SUDEP incidence rates and risk factors.^9^ Age has since been demonstrated in multiple studies not to confer differing rates of SUDEP risk.^10^, ^11^

Majority of PLWE in Uganda experience frequent seizures, which is worsened by anti‐epileptic drug non‐adherence.^12^, ^13^ Whereas seizure control seems the easiest and most attainable way of reducing the risk of SUDEP, there are no clear additional prevention strategies nor guidelines to inform, counsel, and guide PLWE and their caregivers about SUDEP in Uganda. Epilepsy care providers determine the discussions regarding SUDEP with their patients; however, it is not clear as to whether SUDEP is discussed. This lack of discussion might also be influenced by lack of knowledge regarding SUDEP among epilepsy care providers. Therefore, we set out to assess knowledge and experience regarding SUDEP among PLWE and their care providers. We examined the frequency of SUDEP discussion, reasons for discussing and not discussing SUDEP, and asking the healthcare providers about known SUDEP risk factors.

## 2 METHODS

This descriptive cross‐sectional study was carried out at Mulago National referral and teaching hospital in Kampala, Uganda, between August 2017 and February 2018. Mulago is the national referral teaching hospital for Makerere College of Health Sciences and runs both a neurology and mental health outpatient clinics. The two clinics serve as a secondary and tertiary referral centers for patients with epilepsy for the districts surrounding the hospital. The epilepsy patients usually receive attendant care and routine drug refills.

Forty‐eight (48) adults with epilepsy attending the clinics were consecutively enrolled. Patients with a diagnosis of epilepsy (ie, at least two unprovoked stereotyped afebrile seizures with eye witness corroboration with/without supportive interictal electroencephalographic findings) were approached for potential recruitment. Seizures were defined using the 2017 International League Against Epilepsy (ILAE) classification.^14^ Participants provided written informed consent. Those who could not provide reliable history or could not communicate, respond to questions, had mental retardation or dementia, and had no attendant available were excluded.

Eligibility criteria for care providers were providing routine care for patients with epilepsy and drug refills or attending to their attendant concerns within these clinics. These included neurologists, psychiatrists, and assistant physician care providers (also called clinical officers in Uganda). Assistant physician care providers receive a post‐secondary diploma and are involved in patient care and prescribing medications. We sent the pretested study questionnaire to the eligible healthcare providers identified who met the eligibility criteria and those who accepted to participate in the study, provided written consent, filled the study questionnaire (Supporting Material S1), and returned it to the study team.

### 2.1 Measures

Participants responded to a pretested study questionnaire comprising socio‐demographic data that included age, sex, marital status, highest formal educational level, employment status, ethnic group, religion, and selected disease‐related variables such as epilepsy type. The study questionnaire was pretested using a group of health workers who were not providing epilepsy care who considered the question form, wording, and order and also help us to determine whether the questionnaire is understandable by study patients and health workers.

Medical records were reviewed to extract additional clinical information pertaining to the date of initiation of anti‐epileptic drugs (AEDs) and type of therapy (poly‐therapy or mono‐therapy) for PLWE. The questionnaire also explored the understanding of SUDEP, frequency of discussions, reasons for and not discussing SUDEP, etc The study questionnaire for epilepsy healthcare providers included information on demographics regarding the healthcare provider including the type of practice they work in, whether they have received any additional training in epilepsy or clinical neurophysiology, years they have been in practice, and an average number of epilepsy patients they follow in a year. We also assessed their knowledge about SUDEP by identifying known SUDEP risk factors. An arbitrary knowledge score of having two or more correct responses was determined. This score is not validated, but the responses were determined based on the currently available literature.

## 3 RESULTS

### 3.1 PLWE

Over half of the study participants were male 54.2% (26/48). More than half, 64.6% were unemployed and 54.2% attained up to primary or no education. Regarding epilepsy knowledge, nearly 92% (44/48) of PLWE wanted to know everything regarding the disease, while 4.3% wanted reasonable amount of information, and only one person wanted minimal information. The mean duration (SD) in years for the epilepsy among the study group was 14.5 (12.4) years. See Table 1 showing the demographic characteristics of the PLWE.

**Table 1:** Socio‐demographic characteristics of people living with epilepsy

Characteristics	(N = 48)	
Age		
Median (IQR)	25	(19‐34)
Education Level		
None	2	4.2
Primary	22	45.8
Secondary	22	45.8
Higher (diploma, university)	2	4.2
Employment status		
Employed	17	35.4
Unemployed	31	64.6
Marital status		
Single	39	81.3
Married	6	12.5
Divorced	3	6.3
Duration with epilepsy		
≤5 y	9	18.7
≥5 y	39	81.3
Epilepsy type:		
Generalized	42	87.5
Partial	6	12.5
Seizure frequency over the last year		
No seizures	10	20.8
1‐9 episodes/y	20	41.7
10‐20 episodes/y	3	6.3
>21 episodes	15	31.3
Medication type		
Mono‐therapy	9	18.7
Poly‐therapy	39	81.3

Sixty‐five percent (31/48) reported that PLWE had a higher risk of sudden death compared with the general population. When asked whether they have ever heard of a condition called sudden death in epilepsy, 56% (27/48) of PLWE reported to have heard about SUDEP. Half reported they had ever heard about someone who suffered from SUDEP. About 69% wanted to know about SUDEP during the subsequent visits compared with 21% (10/48) who preferred to have the details during the first consultation with the medical doctor. Among those who had heard about SUDEP when asked about their sources of SUDEP information, 7 reported nurses, 5 family member, 4 from a friend, 4 from a general practitioner and one from an emergency care doctor.

**Figure 1:**
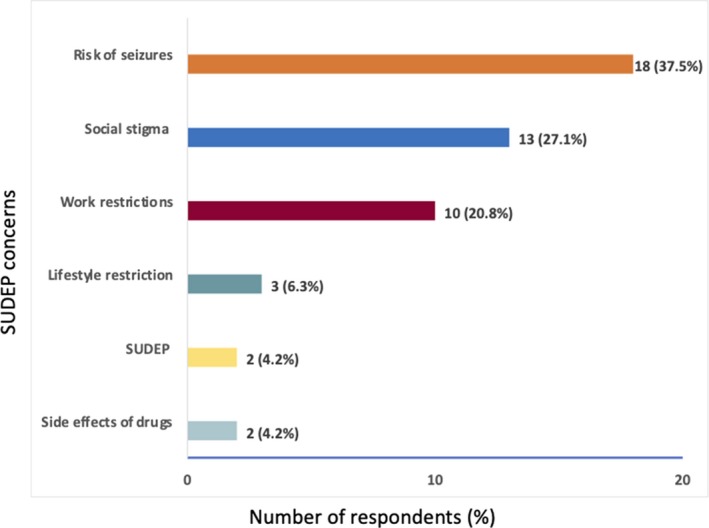
Shows the concerns regarding epilepsy among PLWE. Majority of the study participants were concerned about the risk of a seizure occurring

Only 4.2% (2/48) PLWE were reportedly concerned about SUDEP, and majority of PLWE were concerned about the risk of seizures, social stigma, and work restrictions. See Figure 1, showing the concerns in relation to epilepsy.

### 3.2 Healthcare providers

Twenty‐five provider respondents met eligibility criteria, and 20 completed the study questionnaire for a response rate of 80%; (1 neurologist, 3 psychiatrists, and 16 (80%) assistant physician epilepsy care providers). Table 2 shows characteristics of care providers.

**Table 2:** The characteristics of the epilepsy healthcare providers

Characteristics	All	
	N = 19	%
Profession		
Adult neurologist	1	5.3
Additional training in epilepsy	12	63.2
Additional training in neurophysiology	1	5.3
Academic practice	5	26.3
Years in service		
≤5	12	63.2
>5	7	36.8
Patients seen with epilepsy per year		
1‐10	5	26.3
11‐20	4	21.1
21‐50	3	15.8
51‐100	3	15.8
>100	4	21.0
SUDEP risk factors correctly identified by health workers (Yes only)		
Treatment with 3 or more AEDs	11	57.9
Lack of AED therapy	15	78.9
Electrocardiogram showing QTc	4	21.1
Recent generalized tonic‐clonic seizures	6	31.6
Nocturnal seizures	9	47.4
Childhood‐onset epilepsy	11	57.9
Adults patients with probable or definite SUDEP		
0	15	78.9
1	2	10.5
2	2	10.5
Child patients with probable or definite SUDEP		
0	16	84.2
2	3	15.8

One was excluded due to incomplete questionnaire. The majority (68.5%) had received additional training in epilepsy or neurophysiology. Only 26.3% were in academic clinical practice setting while majority were practicing clinicians. Of these, only 15% (3/20) of healthcare providers reported discussing SUDEP more than half of the time with their patients with epilepsy/caregivers while 85% (17/20) never discussed it.

Eleven percent reported that they discuss SUDEP with people living with epilepsy at the time of diagnosis while 5% when they consider a patient to be at high risk.

The main reasons for not discussing SUDEP were not knowing enough about SUDEP (89.5%) and no adequate support network available (15.8%). See Table 3 showing reasons healthcare providers do not discuss SUDEP with patients or caregivers. Among the healthcare respondents that discussed SUDEP, depression, anxiety, and distress were reported to be the commonest experienced negative reactions following a discussion of SUDEP with their patients.

**Table 3:** Reasons healthcare providers do not discuss SUDEP with patients/caregivers

Reasons	All	
	N = 19	%
Patient is at minimal or no risk.	4	21.1
The information could affect the patient quality of life.	2	10.5
The patient lacks an adequate support network.	3	15.8
I do not have sufficient time to discuss SUDEP during an office visit	2	10.5
Information is available through other sources	1	5.3
SUDEP is so rare and the risks of discussion outweigh the benefits	2	10.5
I do not know enough about SUDEP	17	89.5

Nearly half of the epilepsy healthcare respondents (47%) did not have adequate knowledge regarding the known SUDEP risk factors which we defined as SUDEP knowledge scores of ≤ 2 correct answers.

## 4 DISCUSSION

These study findings highlight gaps in knowledge regarding SUDEP in Uganda among the healthcare providers and PLWE/their caregivers. Attitudes and practices among PWLE and providers may also limit health behaviors and clinical care delivery that have potential to minimize risk for SUDEP. Like healthcare providers in the high‐income countries (HIC), healthcare providers in Uganda do not generally discuss the risks of SUDEP with their epilepsy patients or the caregivers.^15^, ^16^, ^17^ Only 15% of the healthcare providers reportedly discussed SUDEP.

The Report of the Guideline Development, Dissemination, and Implementation Subcommittee of the American Academy of Neurology and the American Epilepsy Society recommends that SUDEP should be discussed with patients with epilepsy and their caregivers and they should be informed of their risks. For persons with epilepsy who continue to experience GTCS, clinicians should continue to actively manage epilepsy therapies to reduce seizure occurrences and the risk of SUDEP while incorporating patient preferences and weighing the risks and benefits of any new approach.^9^

Majority of epilepsy healthcare providers within Mulago hospital did not discuss SUDEP with patients with epilepsy or their caregivers. The healthcare providers reported that when they felt that PLWE was having a high risk, then discussion is initiated especially among those who were missing anti‐epileptic therapy or receiving three or more AEDs and had childhood‐onset epilepsy. However, this might have been influenced by the reportedly lack of sufficient information among the healthcare providers to sufficiently address the questions and expectations by the PLWE. Studies in randomized controlled trials of add‐on AED therapy in patients with intractable partial epilepsy shows that controlling the frequency of seizures may be the most effective way to reduce seizures and PLWE who received appropriate effective doses of adjunctive AEDs had a sevenfold reduction in SUDEP rates compared with those receiving placebo.^18^ Majority of our patients still suffer frequent seizures due to irregular supply of AEDs and lack of out of pocket to purchase these drugs, and policy strategies are needed to address this. Also, a concerted effort is needed to increase educational campaigns targeting the knowledge gaps among both the epilepsy care providers and PLWE. Empowering PLWE and providing them with the adequate information helps asserting themselves and getting the correct information regarding their illness. Probably equipping epilepsy care providers with the required information may make them comfortable to discuss SUDEP with their patients.

Among those who discussed SUDEP, depression, distress, and anxiety were reported as the common reactions following a discussion of SUDEP. It is possible that this unsettling reaction among PLWE influences the SUDEP discussion and healthcare providers may choose to delay this discussion. Whereas PLWE reported that they had heard about someone who had suffered from SUDEP, it remains unclear as to whether the person talked about actually suffered from SUDEP as this could not be verified, taking into account the rarity of this condition even in high‐income countries. Majority of the PLWE preferred having these discussions during the subsequent visits, and this may be stemming from the assumption that subsequently seizure frequency which is an important risk factor would be controlled and the person living with epilepsy would psychologically be feeling better to enable this discussion. Development of tailored information and schedules as to when SUDEP discussions can be initiated is urgently needed to guide epilepsy care providers in resource‐limited settings. Further studies on as to when it is best to give information and details regarding SUDEP in PLWE are needed.

SUDEP remains a challenge with majority of epilepsy healthcare providers having insufficient information. Provision of SUDEP‐related information to improve knowledge and perhaps other approaches that can change attitudes (like change champions, role models, or incentives) may have potential to impact practices among both PLWE and clinicians. Most of the healthcare providers receive medical education from symposia, conferences, and topical discussions or presentations targeting these seem worthwhile. Integrating SUDEP discussions during epilepsy seminars with a clear understanding of known risk factors would help in educating epilepsy care providers with sufficient knowledge and equipping them to educate and stratify patient with epilepsy. Through engagements with policy makers and ministry of health to avail sufficient anti‐epileptic drugs to PLWE by reducing drug stock‐outs, we would address issues of seizure frequency and seizure control, hence reducing SUDEP.

One limitation of our study is the relatively limited geographic representation in a sample of convenience subject to selection bias. This may make it difficult to generalize the study finding; however, given the access to a vast population we serve at Mulago and educational resources in our region, we would expect that, if anything, rates of knowledge might be lower in other surrounding regions. Future studies are needed to increase the number of respondents over the country. The second limitation was recall bias, as these were self‐reported questionnaire for the health workers. The third limitation was regarding the evaluation of knowledge of SUDEP risk factors among the healthcare workers; the scoring system we used was arbitrary and un‐validated and may not be reliable to determine this. A validated scoring system may be needed to further explore this.

In conclusion, we found that epilepsy healthcare providers do not discuss SUDEP with patients with epilepsy often and when they do, they do not discuss it with all of their epilepsy patients.

## CONFLICT OF INTEREST

The authors declare that they have no competing interests. We confirm that we have read the Journal's position on issues involved in ethical publication and affirm that this report is consistent with those guidelines.

## AUTHORS' CONTRIBUTIONS

All authors critically read through the manuscript and revised the manuscript for important intellectual content. All authors discussed the results and commented on the manuscript. All authors read and approved the final manuscript.

## ETHICS APPROVAL AND CONSENT TO PARTICIPATE

The institutional review boards (IRB) of Makerere University, College of Health Sciences’ School of Medicine (Rec Ref: 2017‐112) and Uganda National Council of Science and Technology (UNCST), SS4486 approved the study. All participants provided written informed consent.

## Data Availability

All data generated or analyzed during this study are included in this published article.
