# Supplementary material for: Knowledge and attitudes about sudden death in epilepsy among people living with epilepsy and their healthcare providers in Mulago Hospital, Uganda: A cross‐sectional study
Source: Epilepsia Open. 2019 Dec 26;5(1):80–5. doi: 10.1002/epi4.12374 (PMC7049805; doi:10.1002/epi4.12374)
Supplement: Supplementary file 1 [file EPI4-5-80-s001.docx]

**Study questionnaire**

Study ID____________________________

Age at onset of epilepsy: _________ years Sex: M F

District: ___________________ Tribe: _____________________

1. **Marital status:** Single, Married, Divorced (Tick the correct response)
2. **Employment status**: Employed, Unemployed (Tick the correct response)
3. **Education status attained:** Never attended, Primary school, Secondary school, University level (Tick the correct response)
4. **Epilepsy type:** Generalized, Partial (Tick the correct response)
5. **Seizure frequency over the last year:** No seizures, 1-9 episodes/year, 10 – 20 episodes/year, >21 episodes. (Tick the correct response)
6. **Medication type:** Monotherapy, Polytherapy (Tick the correct response)
7. How long have you had epilepsy? _______________years.
8. With regard to your epilepsy, you would generally like to know
9. Everything possible
10. A reasonable amount of information
11. A minimal amount of information
12. Do you think that people with epilepsy have a higher risk of sudden death compared with people without epilepsy? Yes ______ No________
13. If there is a higher risk, would you prefer to know the details?

Yes ________No_________

1. Have you ever heard of a condition called sudden unexpected death in epilepsy (SUDEP)? Yes_____No_______
2. If your answer is yes, where did you get this information from? (single or multiple responses)

Neurologist

General practitioner

Emergency doctor

Internet

Television/radio/newspaper/magazine

Nurse

Epilepsy support groups

Friend

Family member

Other

1. How much information regarding SUDEP would you like to receive?
2. Detailed information
3. Basic information
4. None
5. Whom do you prefer to obtain this information from?

Neurologist

General practitioner

Emergency doctor

Internet

Television/radio/newspaper/magazine

Nurse

Epilepsy support groups

Friend

Family member

Other

1. If you feel that you need to know the details about SUDEP, when do you think this information should be provided?

During the 1^st^ consultation with the doctor

During the second consultation

During subsequent consultations

When seizure control worsens

1. Do you think SUDEP is preventable? Yes_____No_______
2. Are you personally aware of anyone who has suffered from SUDEP (sudden unexpected death in epilepsy)?
3. What concerns you most in relation to epilepsy?

Driving restrictions

Work restrictions

Lifestyle restrictions

Social stigma

Side effects of medications

Risk of seizures

Sudden unexpected death in epilepsy (SUDEP)
